# “We’re all in this together”: patient and public involvement and engagement in developing a new psychosocial intervention for adults with an intellectual disability who display aggressive challenging behaviour

**DOI:** 10.1186/s40900-025-00675-6

**Published:** 2025-03-06

**Authors:** Connor Clarke, Athanasia Kouroupa, Rachel Royston, Angela Hassiotis, Yufei Jin, Vivien Cooper, Robert Daniels, Lisa Grimley, Sue Hay, Louise Marston, Olawole Odeyemi, Ian Penfold, Claire Pullar, Penny Rapaport, Kate Sanger, Adam Southworth, Laurence Taggart, Afia Ali

**Affiliations:** 1 Division of Psychiatry, University College London, London, UK; 2 Challenging Behaviour Foundation, Kent, UK; 3 Patient and Public Involvement and Engagement Group members of the PETAL programme, London, UK; 4 Department of Primary Care and Population Health, Institute of Epidemiology and Health Care, University College London, London, UK; 5 Institute of Nursing and Health Research, Ulster University, Belfast, UK; 6 Unit for Social and Community Psychiatry, Queen Mary University of London, London, UK

**Keywords:** Patient and public involvement and Engagement, Impact, Implementation, Intellectual disabilities, Inclusive, Participatory research

## Abstract

**Background:**

Although there is consensus regarding the added value of adults with an intellectual disability and family carers as Patient and Public Involvement and Engagement (PPIE) members in research studies, there is limited reporting on the practice and impact of their involvement.

**Methods:**

PPIE input was integral to the application process and subsequent research activities in the NIHR-funded PETAL (PErsonalised Treatment packages for Adults With Learning disabilities) programme. We also conducted semi-structured interviews with five researchers/PPIE facilitators and four family carers, and a focus group with three adults with an intellectual disability who are members of the programme advisory groups. The GRIPP2 checklist guided the reporting of PPIE activities.

**Results:**

Thematic analysis identified four overarching themes: (a) Motivation for being a PPIE member, (b) Added value of PPIE input into research (c) Logistics and practicalities of PPIE activities, and (d) Insights and reflections. Family carers highlighted the benefit of giving a voice to adults with an intellectual disability in PPIE activities within research. Both PPIE groups were positive about being able to share their thoughts and feelings with the PETAL research team and making valued contributions to research activities. All stakeholders highlighted the importance of accessible meeting formats to facilitate PPIE activities. They also reflected on how meaningful collaboration could enhance research in the field of intellectual disability. Researchers raised the need for greater diversity within PPIE groups.

**Conclusions:**

Future work should aim to further develop PPIE processes and identify strategies to maximise the diversity and inclusion of adults with an intellectual disability and family carers in research advisory groups.

**Supplementary Information:**

The online version contains supplementary material available at 10.1186/s40900-025-00675-6.

## Abstract (plain language summary)

This study explored the impact of Patient and Public Involvement and Engagement (PPIE) activities in a research programme that aimed to develop and test a therapy for adults with an intellectual (learning) disability who display aggressive challenging behaviour. We held interviews with five researchers/PPIE facilitators, four family carers, and a group discussion with three adults with an intellectual disability, with most involved in the study from the start of the application and after the award.

Four themes emerged:

Theme 1: PPIE members emphasised their commitment to supporting peers and advocating for those less able to voice their needs.

Theme 2: PPIE members valued the implementation of their ideas about inclusive and respectful language and the knowledge exchange in meetings. Researchers valued knowing what research matters most to adults with an intellectual disability.

Theme 3: Despite some practical challenges, everyone found PPIE activities to be inclusive, with accessible meetings enabling engagement in the programme.

Theme 4: All stakeholders valued collaboration between researchers and PPIE members. PPIE members felt their input led to amendments in study documents to better address. All parties expressed greater confidence in co-creating future research.

Overall, stakeholders viewed PPIE activities as impactful and beneficial. PPIE members discussed with the researchers about the presentation of aggressive challenging behaviour in study documents and amendments were made, where appropriate. Areas for improvement included making sure that people from different countries and cultures are included and supporting people to manage research activities.

**Supplementary Information:**

The online version contains supplementary material available at 10.1186/s40900-025-00675-6.

## Background

Patient and Public Involvement and Engagement (PPIE) in research has been endorsed by the UK National Health Service (NHS) since 1996 (formerly known as consumers in NHS research; now INVOLVE; [1]). The National Institute for Health and Care Research (NIHR) defines PPIE in research as the active partnership among funders, researchers and the public to influence and shape research collaboratively (involvement), disseminate findings (engagement) and take part (participation) in research [2, 3]. There are several commonly used terms to describe PPIE contributors, including experts by experience, people with lived experience, patient or carer representatives, lay researchers and members of the public; these terms are often used interchangeably. The term “public” is an umbrella term encompassing service users of health and social care services, future patients, carers, people representing the population of interest from a charity and/or organisation or other relevant stakeholders [2].

People with an intellectual disability are an underserved population who often receive poor-quality healthcare and require greater support for their physical and mental health [4, 5]. An intellectual disability (more commonly called a learning disability within the UK) is defined as *“having a significantly reduced ability to understand new or complex information and to learn new skills with a reduced ability to cope independently which started before adulthood”* [6]. People with an intellectual disability may experience long-term health conditions such as dementia [7], psychosis [8, 9], arthritis, cancer, diabetes [10], and epilepsy [11] at higher rates than the general population. Some of these conditions, if they remain undetected and untreated, may lead to premature, and otherwise avoidable deaths [12, 13]. Based on the recent NHS England mortality initiative report (LeDeR), the mean age of death in adults with an intellectual disability was 63 years old in 2022 [14]. This is around 20 years younger than the general population in England [15].

To better address the support needs and health disparities of people with an intellectual disability, it is imperative to incorporate meaningful PPIE in health and social care research. PPIE input ensures that research priorities align with the real-world needs and challenges faced by the lived experiences and specific support needs of individuals with an intellectual disability. By integrating PPIE into the research process, we can identify barriers to early detection and treatment of long-term health conditions, tailor interventions to improve health outcomes and address inequities in care delivery. Such collaboration is essential for developing solutions that are relevant, practical and impactful, ultimately reducing premature mortality and improving the quality of life for people with an intellectual disability.

**Fig. 1:**
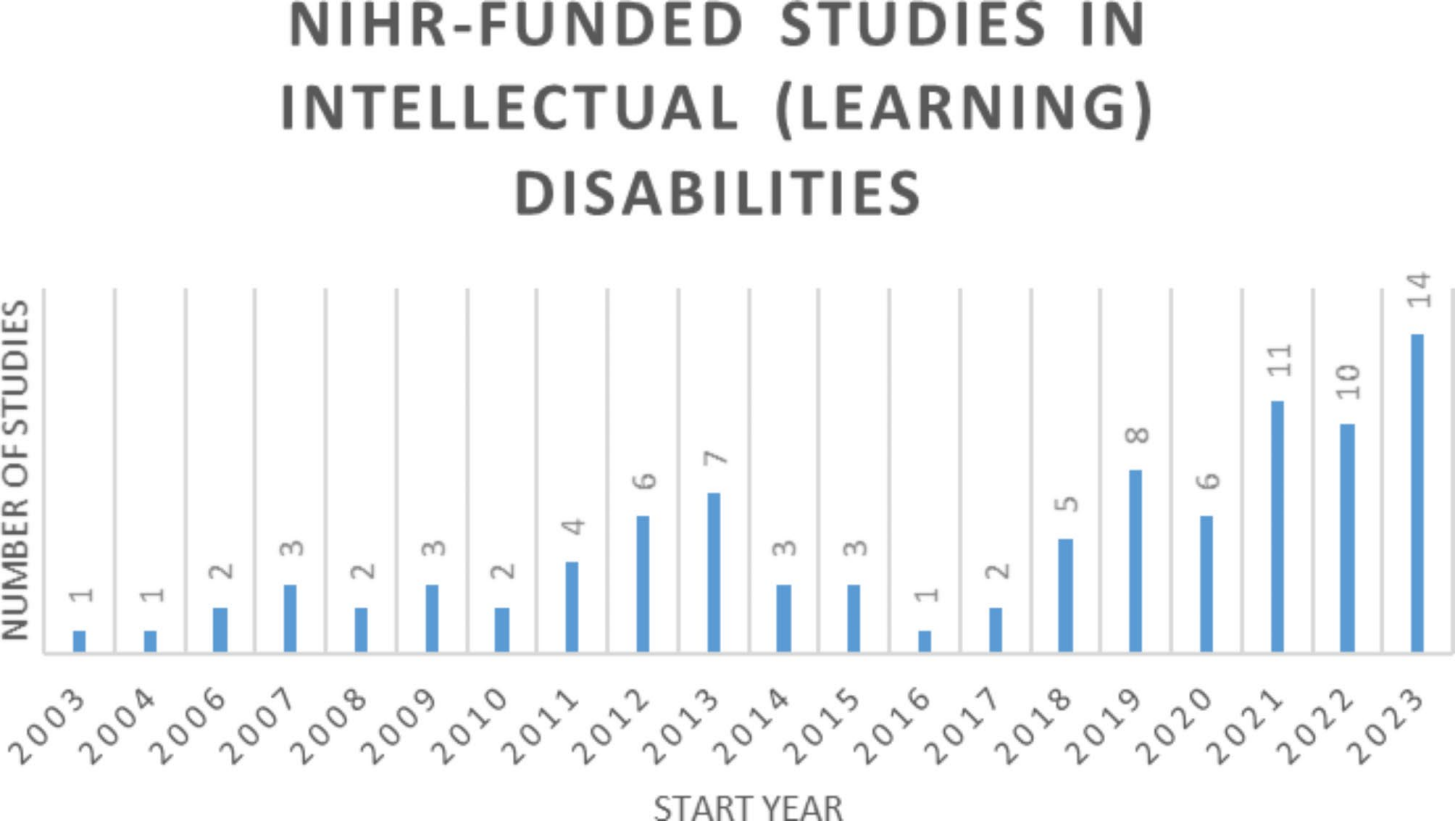
Timeline of NIHR-funded studies* in intellectual disabilities over 20 years *data extracted from NIHR Open Data - Funded portfolio

Indeed, intellectual disability research has steadily attracted more funding over the years. In particular, the first research study in intellectual disabilities was funded by NIHR in 2003 [16, 17]; see Fig. 1). Since then, there have been over 90 NIHR-funded grants in the UK [16, 17]; see Fig. 1) with embedded PPIE input within studies to inform the conduct of research programmes.

Alongside the NIHR, other national and international non-governmental funding bodies (e.g., charities, research councils) equally promote PPIE activities in research. Working in partnership with PPIE members is an essential element throughout the lifecycle of a research study (i.e., to test new drugs, to develop new or adapt existing interventions, to advise on study design, to help with dissemination of results, to improve care pathways etc.). PPIE engagement can facilitate the identification of key gaps within a topic from the perspective of individuals with lived experience and/or address their needs and preferences about the operation of healthcare services with benefits to therapeutic adherence and reduced healthcare costs [18]. In addition, there is increasing interest from the research community to actively work with PPIE members in the design and the conduct of studies to increase the awareness of the public about research and endorse alternative, engaging and accessible ways to disseminate the study findings to the wider public [18]. The value of participatory research is increasingly recognised as having the power to shift the research focus to priority topics for the population of interest [19, 20, 21, 22]. Systematic reviews reported the positive impact of PPIE activities on research including higher rates of participant enrolment and retention [23, 24]. PPIE activities also empower the public whose recommendations are valued and integrated to facilitate the conduct of a research study that leads to benefits for the wider community [25].

Despite these benefits, the inclusion of PPIE activities in health and social care research including intellectual disability research has been slow. Some studies have involved family and paid carers and/or professionals in health and social care research [26] along with adults with an intellectual disability [27, 28]. Of the studies that have incorporated and explored the impact of PPIE activities, just one specified the members’ level of intellectual disability [26]. Some studies sought to understand the experiences of researchers [29], family carers [26] and adults with an intellectual disability [26, 30] as PPIE members in research. These revealed the value of long-standing research partnerships that lead to upskilling and efficiency in meetings, resulting in shared decision-making power between researchers and PPIE members [26, 29, 30]. However, they also noted operational, interpersonal, and contextual challenges (e.g., lengthy steering group meetings, complex research topics being discussed, conflicting personal relationships, funding constraints) that may cause challenges during the course of a research study [26, 29, 30].

In recent years there have been efforts to understand the motivations of researchers, family carers and adults with an intellectual disability for participating in inclusive research [31, 32] and to explore the factors that facilitate or impede this collaboration. Despite this, systematic reporting of PPIE activities, the evaluation of its impact in health and social care research is limited [19, 30, 33]. The development of the GRIPP (Guidance for Reporting Involvement of Patients and Public) checklist in 2011 aimed to address this inconsistency [34]. Since 2011, the original GRIPP checklist has been further revised into the GRIPP2 checklist [35]. The GRIPP2 checklist is the most up to date, international tool informed by multiple community stakeholders (e.g., clinicians, patients, researchers, journal editors) that structures the description and reporting of PPIE activities in research studies. It can be used as a guide to organise PPIE activities at the early stages of a research study and/or act as a quality assurance tool during the later stages of a research study and/or the write up phase of the findings. This study uses the GRIPP2 checklist to evaluate the impact of PPIE activities, contributing to existing research by shedding light on the facilitators and challenges associated with implementing PPIE activities in research involving adults with an intellectual disability and family carers.

### Aim

The current study aimed to systematically explore the impact of PPIE activities by addressing the facilitators and challenges for meaningful PPIE activities in research through evaluating the experiences of different stakeholders involved in an NIHR-funded study, called the PETAL (PErsonalised Treatment packages for Adults With Learning disabilities who present with aggression in community settings) programme. This is a 5.5-year funded programme grant which aims to develop and test a new psychosocial intervention to support adults with an intellectual disability living in the community who display aggressive challenging behaviour. The PETAL PPIE groups (one for family carers and one for adults with a mild intellectual disability) were recruited to provide input to the production of materials relating to the study, to support recruitment, comment on study procedures, and facilitate the interpretation and dissemination of findings. Overall, this study aimed to provide tools to enhance inclusivity and empower participatory research in adults with an intellectual disability and family carers. It provides evidence on the impact of PPIE activities on research processes, outcomes and ethical considerations. The PETAL intervention was co-produced with a group of family carers and of adults with a mild intellectual disability. A few members of the PETAL co-production team were also members of the PETAL PPIE groups. A research paper illustrating the PETAL co-produced intervention is currently within the preparation stage.

## Methodology

### Study design

This qualitative study was conducted 35 months into the PETAL programme to evaluate the impact of PPIE activities, identify the facilitators and challenges to implementing PPIE activities and formulate recommendations for the remainder of the programme. As part of the PETAL programme, we included two advisory groups, one of family carers and the other of people with a mild intellectual disability (self-advocates). We also collected the views and perspectives from multiple stakeholder groups. The latter included a PPIE co-applicant from a national charity for challenging behaviour and a PPIE facilitator for adults with a mild intellectual disability from a self-advocacy group, as well as early career and established researchers working in the PETAL programme. We define PPIE impact as the (positive or negative) effect identified by participants on the research processes and outputs. Under the same definition, we included the impact of PPIE members on researchers/PPIE facilitators involved in the study, and how this contributed to the overall research culture. We were guided by the long form GRIPP2 checklist which is a tool that aids researchers in improving the quality, transparency and reporting of PPIE activities in health and social care research (see Additional file 1) [35].

### PPIE in the PETAL programme

The PETAL programme started in April 2020; two weeks after the announcement of the first national lockdown in the UK due to the COVID-19 pandemic. Therefore, most PPIE meetings have been conducted online via the online platforms Zoom and Microsoft Teams. The PETAL programme facilitated initial training of PPIE members in research processes and encouraged the engagement and attendance of family carers in meetings by having costed replacement carer support to dependent family members guided by NIHR INVOLVE [34].

The national charity Challenging Behaviour Foundation (CBF) acted as contractor for the recruitment of PPIE members of the family carers group. A job description was drawn up and circulated via the CBF national network. The family carer group includes parents from England and Scotland, thus online meetings were the most accessible and convenient choice. A job description for the self-advocates with lived experience of aggressive challenging behaviour was also drawn up and circulated through the networks of the PPIE facilitator to similar services in London. Most meetings with the adults with a mild intellectual disability PPIE group have also been conducted online with an in-person/hybrid meeting in March 2023. There are plans to hold further in-person meetings. All PPIE members are reimbursed for their time on study tasks as per INVOLVE guidelines [36]. Meetings have been facilitated by the programme co-chief investigator, the programme manager and other members of the research team. Before and after every meeting, accessible summaries of the agendas and meeting minutes are shared with PPIE members and their facilitators.

**Fig. 2:**
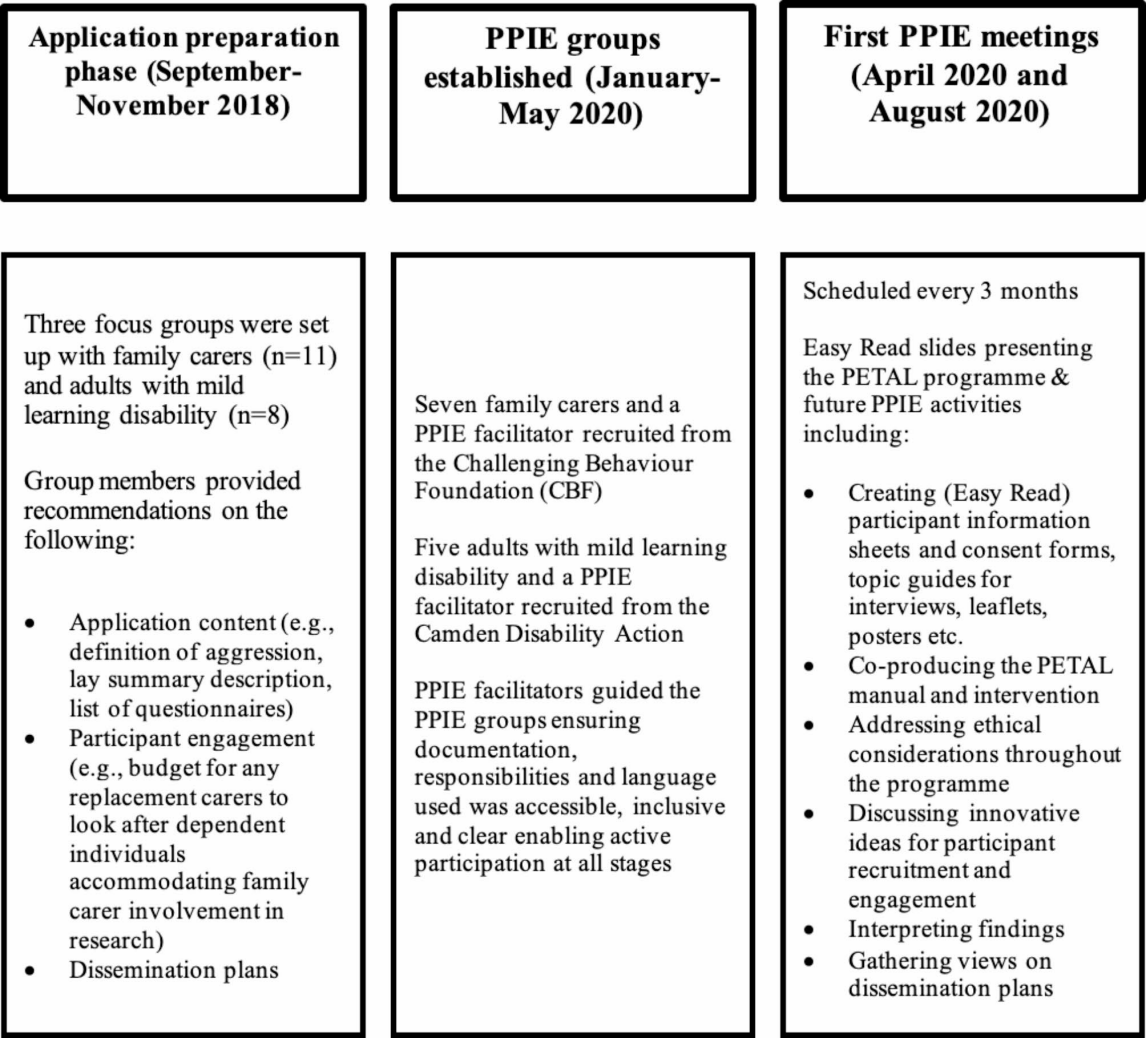
Description of PPIE activities in the PETAL programme

Overall, PPIE activities relating to the PETAL programme took place both pre (2018–2019) and post award (2020) (see Fig. 2). At the application preparation phase, there was PPIE input from local self-advocacy groups and family carers with lived experience responding to an advert requesting input by interested individuals. They provided perspectives on the choice and importance of the research topic/focus, outcome measures, and how they could be involved, if successful in securing funding. In all, during the pre-submission phase, funding from the NIHR London Research Design Service (currently NIHR Research Support Service) funded work with 11 family carers, six adults with an intellectual disability and two autistic people; seven of whom were male. At the start of the PETAL programme, two distinct PPIE groups were formed with support from the CBF and the Camden Disability Action to accommodate the PPIE activities as well as the co-production of the PETAL intervention. PPIE members were mindful of language use that could be perceived as stigmatising to people with an intellectual disability who may display aggressive challenging behaviour. They ensured to mention any such instances in study written materials and presentations including study promotional videos and therapist training documents.

### Participants

The PETAL programme advisory groups included nine PPIE members (five family carers and four adults with a mild intellectual disability) at the beginning of 2023 (vs. 12 PPIE members in 2020). In February-March 2023, 14 participants (nine PPIE members, three researchers and two PPIE facilitators) were invited to take part in interviews, of which 12 participants (four family carers, three adults with a mild intellectual disability, three researchers and two PPIE facilitators) agreed. One family carer and one person with a mild intellectual disability were unable to take part in the current qualitative study due to caring commitments and a scheduling conflict, respectively, regardless of the budget secured for carer support to accommodate family carer involvement in research.

The final sample included three adults with a mild intellectual disability aged 38 to 52 years old (M = 43 years old, SD = 7.81), two were male. One person self-reported as being from a Black ethnic background, and two people as White British. The adults with a mild intellectual disability had been involved with 1–20 research projects in the past including the PETAL programme (M = 7.67 research projects, SD = 10.69).

The five family carers (1 male) were aged 30–70 years old (M = 54.24 years old, SD = 17.17) and all self-identified as White British. Family carers were educated to high school level (*n* = 1) or higher or post-secondary school education (e.g., college, university or technical training; *n* = 3). Including the PETAL programme, family carers had participated in 2–4 research projects as PPIE members. Both PPIE group members were reimbursed for their time and any incurred care costs.

The three researchers (all female) were aged 30–44 years old with one self-identifying as White Other, one as Asian and one as White British. Both group facilitators, two early career researchers who had worked with both PPIE groups for 3 + years and the programme deputy chief investigator were also interviewed about their views. Facilitators were aged 50–60 years old. One facilitator was male and self-identified as White Irish and the other female facilitator self-identified as White British. The female group facilitator was the former chair of the CBF. The male facilitator has many years of experience in facilitating local co-production for services and has worked within research projects with the Chief Investigator for over 10 years. PPIE facilitators were costed as co-applicants in the PETAL programme grant. Therefore, there was no financial compensation for their time in this qualitative study.

### Measures

Three individual topic guides (see Additional file 2), one for each stakeholder group (e.g., family carers, adults with an intellectual disability and researcher/PPIE facilitators), were created by two researchers independent from the PETAL programme (CC and YJ) under the supervision of the PETAL research team. The topic guides were reviewed by the PETAL co-applicant team and the PPIE facilitators. The topic guides were designed to evaluate PPIE impact and included a series of open-ended questions followed by optional prompts, to elaborate and/or clarify specific points. The main topics focused upon the PPIE members’ experiences of their involvement to date, including benefits and challenges, individual contributions to the research and the reasons for their involvement.

### Procedure

Researchers independent of the PETAL programme (CC and YJ) conducted semi-structured interviews with stakeholders in February-March 2023. Individual interviews are among the most common forms of data collection in qualitative research [37] and are particularly useful when exploring areas of novel research topics [38]. These were therefore selected for researchers, PPIE facilitators and family carers to gain a more in-depth insight into the experiences of participating in PPIE activities within the PETAL programme [39]. Two interviews with researchers were conducted in person and the remainder were conducted online via Zoom, lasting for up to 40 min. Focus groups have similar advantages to interviews but also provide an environment whereby participants are surrounded by familiar co-participants and a group context that can be less intimidating than individual interviews [40, 41]. A hybrid online and face-to-face focus group discussion was the methodology of choice for the group of adults with a mild intellectual disability.

### Data analysis

Audio recordings were downloaded from Zoom and were reviewed for accuracy by researchers independent from the PETAL programme (CC and YJ). A reflexive thematic analysis of the data was carried out to identify themes and patterns in the data in accordance with Braun & Clarke [42, 43]. Findings from all stakeholder perspectives were triangulated to provide a holistic understanding of the impact of PPIE activities within the PETAL programme. The data were analysed collectively, comparing the insights across three distinct stakeholder groups to identify shared perceptions, experiences and areas of divergence.

After familiarisation with the data, CC and YJ undertook a process of line-by-line coding of the data using an inductive approach. Once initial coding was completed, the PETAL research team (AK and RR) reviewed and categorised the data. This was an iterative process where discussions about the coding were ongoing until the final formation of themes and subthemes. Data was analysed and reviewed by all members of the research team to ensure a high level of agreement of the developed themes following which the Chief Investigator of the programme (AH) was invited to verify the process of the coding framework. The emerging themes and subthemes were presented to both PPIE groups for comments and revision. The coding framework was organised via the NVivo 14 [44] software.

### Reflexivity

Reflexivity acts as a process of *“introspection on the role of subjectivity”* to ensure rigour and quality in the analysis of research findings [45, 46]. This makes reflexivity a key part of the integrity of the qualitative research process and ensures trustworthiness and accountability is maintained [47]. The lead author of this work (CC) is a white British male who began working on this research study during a placement within an MSc course in the Division of Psychiatry at the University College of London. The lead author of this work continued working as a research assistant once the MSc course had finished. His personal views and experiences may have influenced the analysis of these findings (CC), including his interest in a career in clinical psychology and having family members with an intellectual disability. Consequently, it is likely to have a positive predisposition towards enabling adults with an intellectual disability and family carers.

## Results

Four main themes were identified through the analysis of the data with each theme containing relevant sub-themes.

**Fig. 3:**
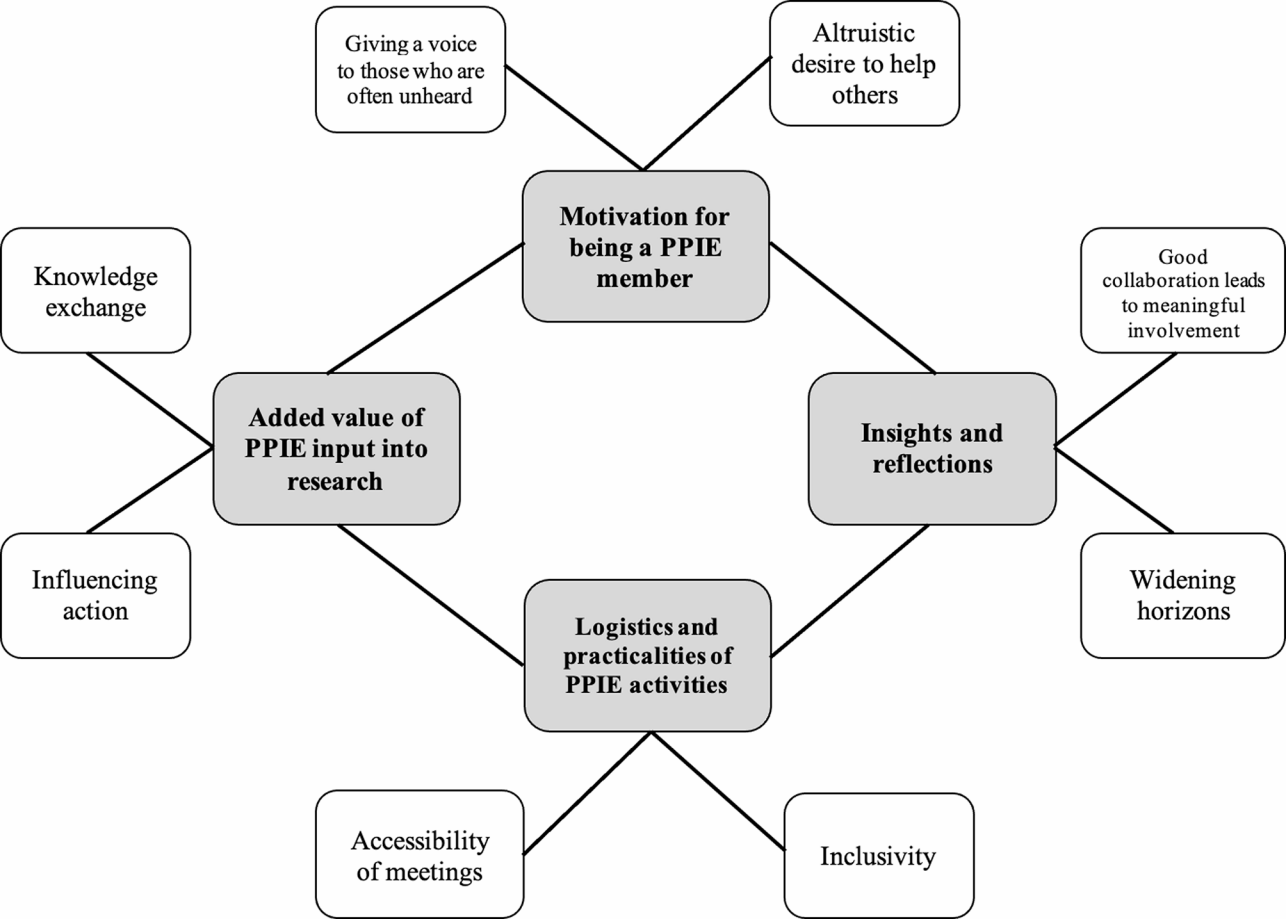
Thematic map of themes and sub-themes

The main themes were: (1) ‘Motivation for being a PPIE member’; (2) ‘Added value of PPIE input into research’; (3) ‘Logistics and practicalities of PPIE activities’ and (4) ‘Insights and reflections’. Each sub-theme is presented with relevant quotations and is reported in further detail. Each of these themes and sub-themes are presented in Fig. 3. PPIE facilitators and researchers at all career stages are labelled as ‘researchers’ to protect anonymity.

### Theme 1: Motivation for being a PPIE member

Motivations for participating in the research programme report data from the two PPIE groups only. The members of the advisory groups were already self-advocates and had lived experience of aggressive challenging behaviour either themselves or in their families. Therefore, by becoming part of the PPIE framework of the programme, members of the two PPIE groups meant that they could generally contribute to research in the field of intellectual disabilities broadly, communicate their views and ensure that we are mindful of the stigma associated with behaviours that challenge more generally and in support of a study that could influence practice. They focused on the importance of giving a voice to people with an intellectual disability who are often unheard and those described as hard to reach. The value of altruism arising from the personal drive to help others, is also described.

#### Sub-theme 1.1: Giving a voice to those who are often unheard

Family carers expressed their motivation to support research and enhance the knowledge and understanding of adults with a mild intellectual disability who display aggressive challenging behaviour. Family carers emphasised the importance of the voices of adults with a mild intellectual disability being heard and how this should influence the direction of research, particularly for those with social and communication needs whose insight is often not well represented.

*“I have a brother who uses aggressive challenging behaviour. He has a severe learning disability so getting his views directly would be difficult so I think there’s definitely an advocacy role for me in terms of sharing some of that experience of his.”* (Family carer 1).

*“Sometimes in a world like we live in today*,* children like [mine’s] voices are lost so I can be that voice.”* (Family carer 2).

#### Sub-theme 1.2: Altruistic desire to help others

Adults with a mild intellectual disability were eager to join the PETAL PPIE group to support others to live more purposeful lives by sharing their personal journeys and experiences.

*“I help*,* because of my experience. I had to share that experience with other people*,* they can have a better life.”* (Adult with a mild intellectual disability 1).

*“You know I was happy to be asked from day one because I like to help others like that have disabilities*,* you know. I like to help out when I can.”* (Adult with a mild intellectual disability 2).

Similarly, family carers described their participation in the PETAL PPIE activities as being driven by a desire to support other families to improve their quality of life and subsequently the well-being of adults with an intellectual disability who display aggressive challenging behaviour. The financial incentives were appreciated but played a minimal role in their decision to participate in research.

*“I work very hard to try and improve the system for people like my son to make sure that their needs are better understood.”* (Family carer 3).

*“I’ve been offered… remuneration a lot of times and that has been…well received… I don’t actually take the payments because I do it voluntarily…I get such a lot of satisfaction out of helping with projects you know like this that’s payment enough for me.”* (Family carer 3).

### Theme 2: Added value of PPIE input into research

Both PPIE groups and researchers shared the opinion that PPIE input helped to add value to the overall study leading to concrete actions.

#### Sub-theme 2.1: Knowledge exchange

The expertise and input from both PPIE groups were valued by the research team. The research team stated that the knowledge and experience of family carers and adults with a mild intellectual disability gave them greater insight and the opportunity to self-reflect upon their own practices and research priorities. For example, some researchers described how their research agenda may often be influenced by their own interests, rather than what might be most important for people with an intellectual disability. It was acknowledged that the PPIE input could enable a re-focusing of future research directions which extended beyond the specific project.

*“It’s opened my eyes to kind of a wider range of perspectives that perhaps I hadn’t thought about in research or my clinical practice so… personally it’s just being kind of more aware of the issues that perhaps I wasn’t aware of and kind of being able to reflect on them and then sort of trying to make a difference.”* (Researcher 1).

*“It’s been really helpful to get their insight and it’s been really invaluable…to help us know what we should be prioritising*,* what’s not as important*,* what should be included and what’s important to them because that’s really what we should be focusing on*,* not what necessarily we think is important.”* (Researcher 2).

The exchange of knowledge as part of the research programme was helpful not only from the viewpoint of the researchers but also by the members of the PPIE groups, specifically around ways in which they could better support those they care for.

*“I learned other things from the other contributors as well because when I watched the videos of them doing their talk or explaining how they supported their family member I thought oh… that’s a great idea I must try that.”* (Family carer 2).

#### Sub-theme 2.2: Influencing action

All stakeholders recognised the important role of both PPIE groups in the design and management of the PETAL intervention. For instance, PPIE members commented on the research design and study processes, reviewed documents, contributed to dissemination and study promotion activities and co-designed with the research team a manualised intervention. PPIE members have also been involved in the training of clinicians delivering the PETAL intervention. Input from both PPIE groups towards this training was not initially planned but it was suggested by the PPIE members and agreed upon by the research team as being a valuable addition.

*“You know it’s… a knowledge that we have to help people to develop and to have the better training manual to help them to develop with their lives.”* (Adult with a mild intellectual disability 1).

*“My most valuable contribution… the interviews that were filmed for the training modules.”* (Family carer 4).

*“Their input makes a difference so it’s absolutely worthwhile and I think the project has been shaped based on their input and feedback on things.”* (Researcher 2).

One of the most important ongoing contributions of the PPIE groups has been the guidance and consensus building relating to the use of terms and language used within meetings and study documentation.

*“The language being used was I felt inappropriate in some cases the use of the word aggressive and treatment were two of the things that kind of touched a nerve because…if you ask somebody…what’s aggressive they see it as a very negative thing most people will if you don’t give them the context and even if you give them the context sometimes they won’t understand unless they’ve had the experience themselves.”* (Family carer 3).

*“For example the phrase aggressive challenging behaviour and it took a very long time to get that point across and come to some sort of compromise that said you know you still gonna use that of course you are but that we could sort of use some sort of disclaimer to distance ourselves from that language as language that we wouldn’t personally use.”* (Family carer 1).

Researchers and the PPIE family carer group members agreed that whilst the discussion of language and terminology often led to differing viewpoints and opinions, agreements were reached on how to move forward in a way that all team members felt comfortable (e.g., by providing statements within documents to explain the use of specific terminology).

*“So there has been times where there’s been disagreements-not disagreements but…just where people have… viewpoints conflict with each other so that makes it then hard for us to decide which way to go.”* (Researcher 2).

*“Eventually the team actually recognised the importance of language more than they had previously perhaps and even though we weren’t able to stop them using those terms they put us a paragraph somewhere in the study that actually summarised… why they were using those terms.”* (Family carer 3).

It was also highlighted that there is limited flexibility to make substantial changes in funded studies but researchers incorporated suggestions, whenever possible.

*“I think it’s important*,* you know*,* at the beginning to say to the PPI groups… this is what’s in this bit and this is why we put it in like this but do you think it needs to change? We’ve often said in the meetings… when the researcher said*,* oh*,* this is what we do*,* this is what we’re doing next.”* (Researcher 3).

*“It has been a little bit tricky to sort of always act so we haven’t been able to act on all the suggestions. I think that’s fair to say that some of the feedback we’ve got we certainly listen to it but the nature of the grant is such that we have certain research questions and certain things needs to be done in a certain way…but certainly we’ve always tried to look at the feedback and we’ve tried to action on the feedback as much as possible”* (Researcher 1).

### Theme 3: Logistics and practicalities of PPIE activities

All stakeholders raised the operational challenges of setting up and maintaining the engagement of PPIE groups throughout the study.

#### Sub-theme 3.1: Accessibility of meetings

Family carers expressed how researchers were supportive and open to making attendance as convenient as possible, offering different meeting dates and times and taking into consideration the caring responsibilities of family carers.

*“Normally…there are…2 or 3 suggestions about timings for meetings and plenty of notice has been given as it’s online… I have been away once but I could still join the meeting online so it wasn’t a problem. You’re never gonna get a suitable time for everybody. But certainly our opinion is asked for about when meetings take place it can be flexible as possible.”* (Family carer 4).

However, one family carer raised that there was limited discussion prior to their involvement about what would work best for the entire PPIE group.

*“There wasn’t enough discussion… probably about… maybe halving the group and having some in the daytime and some on an evening.”* (Family carer 1).

Meetings started during the lockdowns of the COVID-19 pandemic meaning these were mostly held online via Zoom. Most family carers perceived online meetings as convenient and for the most part, easily accessible, particularly for those living outside London.

*“I’m not sure it was ever actually discussed that it would be in any format other than online but yeah I think my preference… is online not just for work reasons but also for accessibility reasons and things like that so it’s definitely been helpful for me that it was held online.”* (Family carer 1).

The format of meetings may not have been entirely what some family carers originally anticipated when they joined the study. However, this change in expectations was beyond the research team’s control due to the start of the COVID-19 pandemic in March 2020.

*“I mean it was just described as giving a lived experience as a family carer of somebody who has aggressive challenging behaviour…so it was pre-pandemic when it was set up so we didn’t know that all the meetings were going to be online at the beginning…so yeah from that point of view it’s not been quite as I expected.”* (Family carer 4).

Similarly, one family carer was not aware of the extent of the commitment that was necessary to be involved in this research. This meant that they were not always able to dedicate the full amount of time required to take part in meetings.

*“I don’t think I had an understanding beforehand of how much of a commitment it was gonna be how frequent the meetings would be which had I had that I think I would have known that I couldn’t give the time required.”* (Family carer 1).

Some of the researchers reported the use of virtual meetings limited the opportunity for PPIE members to connect and build relationships with the research team and their peers in order to form a more cohesive group.

*“it’s been more difficult to perhaps establish those relationships online whereas… you would ordinarily… have a face to face meeting and you might have some tea and coffee and cakes and that might sort of help you to… build relationships and… that’s been missing… so it’s taking a while… to kind of build relationships and build trust.”* (Researcher 1).

Finally, one adult with a mild intellectual disability reported that the use of pictures and easy-to-understand language in the study documents, in meetings and meeting minutes facilitated their contribution to the PETAL programme during the different phases of the study and made the meetings accessible for them.

*“I think that… using the pictures and the wording that’s been good.”* (Adult with a mild intellectual disability).

#### Sub-theme 3.2: Inclusivity

Family carers reported there was a good range of perspectives within the group in terms of caregiving roles. Family carers stated that they were provided with opportunities and encouragement to share their opinions and perspectives.

*“I think overall as a sibling I have a slightly different perspective… so for me it’s about making sure that there’s that sort of perspective that understands the needs of siblings as a different need and as a separate need but also the understanding and insight of siblings as slightly different and slightly separate to those of parents because it is a different relationship.”* (Family Carer 1).

However, the need for more diverse groups (e.g., sex, ethnicity) in future PPIE work was highlighted.

*“I’m not entirely sure whether with the carer group whether we’ve necessarily got full kind of representation from kind of lots of different backgrounds and kind of ethnic groups. The carer group is largely female and kind of largely sort of white…I think maybe thinking about how we can improve the kind of inclusive kind of diversity of carers to make sure that we catch kind of a wider range of perspectives I think is something that we could certainly work on and improve.”* (Researcher 1).

### Theme 4: Personal insights and reflections

All stakeholders acknowledged the value of working together (with researchers and PPIE members) on this project which strengthened their confidence and enhanced their skills to shape and co-create research studies. Researchers and PPIE members described their determination to support the involvement of members of the public in research and facilitate the implementation of PPIE activities to enhance the quality of the PETAL programme. For the researchers, working with PPIE groups provided a constant reminder of the broader picture and true purpose of the research.

#### Subtheme 4.1: Good collaboration leads to meaningful involvement

The research team and both PPIE groups recognised the importance of good collaboration to advance the study and make an impact on the lives of adults with an intellectual disability and their family carers. Adults with a mild intellectual disability and family carers indicated their views were genuinely valued by the research team throughout their involvement.

*“I thought that not only our voices were heard but they were listened to… the team made sure that they knew why we were making those comments-why I was making those comments because that’s so important you know understanding the why’s and the wherefores and so on and lived experience actually gives you the means with which to do that if you can only have time to be heard. And I felt that we were given time to be heard*,* and space to be heard so it was quite a positive experience.”* (Family carer 3).

*“It’s like they are taking our views seriously*,* and… I wouldn’t be a part of it either if they didn’t take us seriously because… as we’ve put some things… that need to be an easy read and they took it back and changed it so yeah*,* they are listening to us.”* (Adult with a mild intellectual disability).

*“We’re all in this together*,* we’re all working together*,* and everyone’s equally valued*,* and so it’s not just the voice of the researchers going forward*,* it’s a… collaborative voice.”* (Researcher 3).

#### Subtheme 4.2: Widening horizons

Some adults with a mild intellectual disability described their scepticism and hesitation to join the PPIE group initially. However, once they settled in, they reported feeling more comfortable, participating and contributing easily within the group’s sessions.

*“I feel more comfortable now than at the start because at the start I wasn’t exactly sure whether I actually wanted to take to take part”* (Adult with a mild intellectual disability).

Similarly, members of the family carers group expressed their satisfaction once they realised their comments and ideas were being incorporated into the study. Family carers, in particular, referred to the power of accessible, simple and inclusive presentation of practices by researchers. This made family carers feel more confident in their abilities to make meaningful contributions that would have an impact on the research.

*“Not all of us feel confident that we can come and do this but I think if something is put in that format and it’s not frightening and it doesn’t look like well could I do it? They think well you know what I could take part in that study cause I would like my daughter to achieve that or my son to achieve that.”* (Family carer 2).

One researcher emphasised the increased willingness and commitment of family carers and/or adults with a mild intellectual disability to undertake more roles and responsibilities and learn new skills through their involvement in new tasks.

*“I think maybe the only thing I could say is just how involved our PPI group is and how willing they are to take on all the opportunities that we have to offer so when we said-asked them if they would be interested to take part in the training*,* to like to deliver training to therapists without hesitation they were really interested*,* really keen*,* really on board.”* (Researcher 2).

Finally, researchers were grateful for the powerful and constant reminders expressed by PPIE group members, about the reason(s) research is important and its role within the wider context to keep the research on track.

*“My experience has been really positive and I think they have given so much insight that we wouldn’t have had otherwise. It’s been really helpful and also when you meet with the PPI members*,* it reminds you what you’re here for and the like whole motivation behind what we’re trying to do… you get reminded of that whenever you meet with the family carers and with the service users and just kind of brings you back to… the real purpose of what we’re trying to do.”* (Researcher 2).

## Discussion

This study provides support for the impact of PPIE activities on the PETAL programme and captures the experiences and perspectives of individuals with lived experience including adults with a mild intellectual disability, family carers and the PETAL research team. As discussed, the NIHR defines PPIE in research as the active partnership among funders, researchers and the public to influence and shape research collaboratively (involvement), disseminate findings (engagement) and take part (participation) in research [2, 18]. This study was particularly concerned with the involvement and engagement part of PPIE as it explored the experiences of PPIE members in relation to the activities they took part in. PPIE activities have been embedded within the PETAL programme at all stages of the study, starting from the development of the grant proposal (2018; involvement) until now (2024; involvement and engagement). The study is ongoing, with an active role of PPIE members in supporting its progress in recruitment, study promotion, intervention delivery to participants, intervention training to healthcare professionals and commenting on the evidence presented at meetings.

Our findings indicate that the experiences of all stakeholder groups have been overwhelmingly positive. Existing literature has already highlighted the importance of involving adults with an intellectual disability and family carers in research [26, 48]. Family carers and adults with a mild intellectual disability with previous PPIE experience described beneficial outcomes in their self-esteem and confidence because they identified that PPIE provided them with a platform to share their expertise which may lead to changes in research that improve their experience of PPIE itself [26, 49, 50]. The benefits of PPIE activities in research, to service users (including adults with a mild intellectual disability and family carers) and the wider community appear evident [19, 48, 50, 51, 52]. These include setting the research agenda, improving interventions, identifying the rationale behind the selection of outcome measures, reviewing recruitment practices, collecting informed consent, facilitating participant retention throughout the study conduct and proposing actionable strategy implementation plans. It also ensures research priorities reflect what is important to the target population [53]. Both PPIE groups were invited to provide their input towards resources and training materials for clinicians delivering the PETAL intervention across multiple NHS sites in the UK. Family carers also supported the delivery of PETAL intervention training, which has been incredibly well received by PETAL therapists. PPIE groups were also invited to give suggestions for videos and study promotion materials and were offered to take part in short videos that were sent out to potential participants, in which they discussed the significance of the study. These examples highlight the tangible outcomes for both PPIE groups in the design and delivery of the study.

Nonetheless, a small number of family carers and some researchers reported logistical and practical challenges relating to the accessibility of online meetings because of the COVID-19 pandemic. It was also recognised that being full-time carers made it challenging at times to take part in meetings especially if alternative care arrangements could not be made. Flexibility around the timings of meetings was seen as an important gap in such circumstances. This issue consistently presents itself as a barrier to participation in studies involving PPIE groups and may create a barrier for some family carers and/or adults with an intellectual disability to get involved with research [54, 55, 56]. The NIHR have suggested that researchers consider a hybrid model whereby face-to-face meetings are combined with remote meetings [36, 57]. The decision to hold meetings should take into account the comfort and convenience of all participating members and thus should be made collectively [36, 57]. The value of meetings being undertaken in person has been highlighted in a recent study by Jones and colleagues [58] in which public contributors’ and PPIE professionals highlighted the difficulty of establishing relationships with researchers and other members of their PPIE group when meetings were remote and how face-to-face interaction was a crucial factor for their involvement in research more generally. It is possible that our PPIE members were overwhelmed to start with and found it challenging to have too many online meetings. Depending on the project needs and costs and the variation in geographical locations of PPIE members, there may be a requirement for having different approaches to how those meetings run, for example including frequent breaks, break out rooms and appropriate facilitation. Where possible an in-person meeting once a year in a long running study may alleviate some of these negative feelings and foster inclusion and ownership of the study. Nonetheless, even for those PPIE members who did not make use of online meetings, the flexibility of remote options was seen as beneficial due to geographic constraints or work/caring responsibilities.

The PETAL programme, in particular, costed care replacement for family carers with caring responsibilities and accommodate their contribution to PPIE activities (i.e., meeting attendance, document review, training delivery) in the PETAL programme [36]. Although this initiative was implemented to enable inclusivity and engagement in research, it received little attention from our family carer PPIE group. It is likely that family carers are reluctant to embrace this initiative due to operational and contextual reasons. For example, family carers might be discouraged to get involved in another administrative task or they are not well informed of this alternative caring model in research. It is also important to highlight that depending on the family culture and their background, they might feel uncomfortable (e.g., cultural barriers, stigma) to claim compensation for such a task or equally be concerned about the relationship of the new carer with the person with an intellectual disability for a short time.

Despite the noticeable increase of PPIE activities in the literature and the UK legislation that actively encourages PPIE in research, there are still areas for improvement. The experiences expressed by the researchers in this study indicated there were times when it was challenging to implement and/or incorporate suggested changes (i.e., research questions) which could sometimes result in conflict and/or disengagement from participation. This challenge had also been reported by Paul & Holt [50], who found that PPIE suggestions may sometimes be impractical or unfeasible within the context of a specific study and can lead to tensions between the PPIE members and researchers. Such conflicts must be handled sensitively to ensure that strong collaborations and working relationships are maintained [59]. Though we have limited flexibility in making changes to a funded study, especially changes to the topic focus and the study protocol, less significant changes can be made when appropriate, dependent on resources and cost. We recommend setting up a framework for PPIE as a study starts and obtaining consensus from members. Discussions should take place with PPIE members so that they understand which parts of the study will contribute to. For example, checking documents for ethical review and commenting on language, presentation and accessibility; acting as co-researchers and participating in the data analysis and interpretation. Providing training at different times during the study could further aid PPIE members to feel empowered in their engagement with research and the researchers.

Researchers, also, highlighted the lack of diversity within PPIE groups which requires further attention by both researchers and self-advocacy organisations to ensure inclusive, personalised and holistic care for adults with an intellectual disability in the future. This lack of diversity remains a barrier to improving (research and clinical) practices since individuals from ethnic minority backgrounds, fathers or male carers and those of lower socioeconomic status, rarely participate in PPIE research [55, 60]. The inclusion of adults with a more severe intellectual disability has been explored by Bunning and colleagues [61] through the use of Talking Mats when conducting interviews in relation to television watching habits. Such techniques should continue to be explored among researchers in order to widen the inclusivity and diversity among adults with an intellectual disability who take part in PPIE research. For example, while the NIHR encourages the use of accessible and inclusive practices in research (e.g., EasyRead formats) for children, individuals with an intellectual disability and other cognitive impairments, these documents are not part of the application submission process although they are considered outputs throughout a study.

### Strengths and limitations of this study

This study expands on current research by providing insights into the experiences of PPIE members in research with adults with a mild intellectual disability. It integrates the views of adults with a mild intellectual disability, family carers and researchers involved in a funded research study and presents rich data on the experiences and opinions of people with lived experience and researchers. Conducting this work has allowed the researchers to forge stronger links with PPIE members and ensure their involvement is meaningful. Interviews were conducted by two independent researchers, with no involvement with the PPIE members at the time of the interview, limiting response bias and allowing participants to freely express their views and experiences about the impact of their contribution.

This study also has limitations. The majority of our PPIE members have been involved with research for many years and this is likely to create an element of bias as our members may have a greater interest, understanding and knowledge of research. There was also limited ethnic diversity within our PPIE groups, particularly for the family carers’ group. However, efforts were made to ensure members were from a broad geographical area of rural and urban locations within the UK. In addition, the voice of adults with more severe disability/minimal verbal ability were not directly collected and were instead represented through opinions of their caregivers. Therefore, this may not be representative of all adults with a moderate-severe-profound intellectual disability.

It has also been suggested that focus groups can raise potential challenges for adults with a mild intellectual disability, particularly in relation to their ability to respond and engage to questions and conversation [62]. However, this potential issue can be mitigated through the use of facilitators who help to encourage free-flowing conversation alongside other individuals who may share similar experiences, thus helping members feel more comfortable to share their own experiences [63]. We believe these considerations during the running of the focus group ensured that PPIE impact was still accurately measured and therefore improved our understanding of the experiences and insights of the group [62, 63].

Finally, there are alternative methods of measuring the impact of PPIE that may offer alternative aspects of PPIE impact. For example, the Public Involvement Impact Assessment Framework Guidance (PiiAF) tool [64] measures PPIE impact by assessing the approach taken by researchers to involve PPIE group members within the study, values associated with PPIE, the focus of the research and practical issues and then guides researchers to develop a plan to help assess the PPIE impact. Compared to the GRIPP2 checklist tool, the PiiAF appears to be a more complex tool. But it is possible that its flexibility in the way in which it can be adapted to a specific research context [65], raises the possibility that an alternative measuring tool might more accurately capture the impact of PPIE within the PETAL programme.

### Implications for including PPIE activities in future research

To enhance participation and create more valuable connections between PPIE members and clinical and research teams, we propose that adults with an intellectual disability are accommodated through reasonable adjustments and the use of inclusive methods where possible and relevant. This could include the use of talking mats and/or play game activities which in turn requires significant support and input from (family or paid) carers and/or other facilitators [61]. There is also a need for adequate training for researchers to gain the necessary experience to involve PPIE groups within their practice and to feel confident leading PPIE advisory groups. The researchers involved with PPIE in the PETAL programme had more than three years’ experience working with individuals with lived experience within the intellectual disability field, but this may not be reflective of all research studies and teams. Similarly, the PETAL PPIE members all had previous research experience. Yet, engaging individuals with lived experience who have less experience is imperative to gain a comprehensive range of perspectives, and training must be offered to PPIE members when they join a study. Several training programmes [66, 67] have been developed to support people with an intellectual disability to participate in research but these are not readily available. Running PPIE groups requires the creation and maintenance of long-term partnerships with third-sector organisations (e.g., charities) and/or members of the public based on trust, transparency, and honesty. These relationships need to be prioritised and research teams should consider in advance how they can support these organisations so that relationships are mutually beneficial.

## Conclusions

The current study explored the impact of involving adults with a mild intellectual disability and family carers within a research programme aiming to develop and test an intervention to support adults with an intellectual disability who display aggressive challenging behaviour guided by the GRIPP2 checklist. The study highlights there is scope to develop and streamline PPIE processes further by broadening diversity and ensuring that involvement is accessible to all, ultimately making PPIE activities more meaningful. This study informs wider PPIE research within the field of intellectual disabilities and future studies should continue to place emphasis upon highlighting the importance of involvement by people with lived experience and working towards holistic, inclusive and quality PPIE activities that can be translated into actionable strategy implementation plans.

### Lived experience commentary

“We are happy with the findings in this report. The project helps people to have a voice, and it can be very difficult for carers and even more difficult for people with learning disabilities to have their voices heard. That is why it is so important to be a part of this project that makes a difference to people. We feel our input has changed the way the research has been run, for example, when putting together the study materials and therapy. Our family carer group has also helped to deliver the PETAL therapist training, something that was not originally planned, and this has been very well received by therapists. We agree that our involvement has made a difference to help researchers think more about the language they are using and has made the work more accessible for us and others.

Due to COVID-19, there were some changes made to the way meetings were run, and this meant we had to adapt and get used to new technologies, but this also meant less time for travelling and our involvement interfered less with our other work and caring responsibilities.

We also really value the fact that we have had the opportunity to meet altogether (family carers and service users) for some of our meetings, and this is something we think is quite rare and creates a joint approach where everyone’s views are heard.

Whilst PPIE groups tend to be small and it can be challenging to get a representative and diverse group of people involved, we agree that having people from ethnic minorities represented in these groups is important and is something to think about for future research. We would also like the opportunity to have more in-person meetings.

Overall, we feel that the findings from this report are reflective of our experiences and will be important to encourage and support future PPIE in research that is meaningful, valuable and worthwhile.”

## Electronic supplementary material

Below is the link to the electronic supplementary material.

Supplementary Material 1

Supplementary Material 2

## Data Availability

No datasets were generated or analysed during the current study.

## Abbreviations

PPIE: Patient and Public Involvement and Engagement
NHS: National Health Service
NIHR: National Institute of Health Research
GRIPP: Guidance for Reporting Involvement of Patients and Public
PETAL: PErsonalised Treatment packages for Adults With Learning disabilities
CBF: Challenging Behaviour Foundation
