# Supplementary material for: “We’re all in this together”: patient and public involvement and engagement in developing a new psychosocial intervention for adults with an intellectual disability who display aggressive challenging behaviour
Source: Res Involv Engagem. 2025 Mar 6;11:20. doi: 10.1186/s40900-025-00675-6 (PMC11887246; doi:10.1186/s40900-025-00675-6)
Supplement: Supplementary file 2 — Supplementary Material 2 [file 40900_2025_675_MOESM2_ESM.docx]

# Additional File 2: Topic guide for researchers and PPIE facilitators group

Hello! Thank you for your time today. We are going to ask you some questions about your experiences being involved with PPIE groups within the PETAL study. All the information we collect will be kept confidential, and your answers will be anonymised. There is no pressure to answer every question. Please only answer questions you would like to answer.

1. Could you please tell us about your experiences of being a PPIE facilitator within the PETAL study?
2. What do you think has been the most valuable contribution made by the group?
3. What other contributions have been made?
4. What has given you the greatest satisfaction in working with the group?
5. What, if anything, has been difficult or challenging in working with the group?
6. Where, if at all, have you experienced any unexpected benefits from working with the group?
7. Have you had any reconsiderations about what you find important within this study and has working with the group challenged your assumptions in any way? If so, how?
8. In what way do you think working with the PPIE group has impacted your use of time, resources and funding?
9. What lessons do you think could be learned from this experience of PPIE?
10. Could you describe how the contributions from the PPIE group were considered by those in a position to act on them?
11. Did involving PPIE groups in this study have an impact on you as a researcher? If yes, how?
12. Is there anything else you would like to add?

We have now come to the end of the interview questions for today. Thank you very much for your time.

# Appendix A: Topic guide for family carer PPIE group

Hello! Thank you for your time today. We are going to ask you some questions about your experiences being involved as a member of the PETAL study Patient and Public Involvement and Engagement (PPIE) group. We would also like to understand if there is anything we can do to improve your involvement. All the information we collect will be kept confidential, and your answers will be anonymised. There is no pressure to answer every question. Please only answer questions you would like to answer.

We will first collect some brief background information that we will use in our reports to summarise the characteristics of the people who have taken part in these interviews. This is standard practice when completing research interviews. Do you have any questions before we start?

**Part 1: Questions about you**

1.What is your gender?

[ ] Male

[ ] Female

[ ] Non-Binary

[ ] Other

[ ] Prefer not to say

2.What is your age?


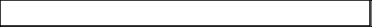


3. What is your ethnicity?

[ ] Asian/ Asian British

[ ] Black/ Black British

[ ] White/ White British

[ ] Mixed

[ ] Other

4. What is the highest level of education that you have completed? P*lease select the highest level of education achieved.*

[ ] No schooling

[ ] Primary school

[ ] Secondary school

[ ] Higher or post-secondary education (college, university, technical training)

[ ] Postgraduate, professional, or doctoral degree

[ ] Prefer not to say

5. How many research projects have you been involved in as a PPI member (including the current PETAL study)?

[ ] 1 project

[ ] 2-4 projects

[ ] ≥ 5 projects

**Part 2: Your views about your involvement within the PETAL study**

2A. I am going to ask you a set of questions now about being part of the PETAL study about why you got involved and how you have contributed, then about the meetings themselves and any improvements we can make.

1. Why did you want to get involved in research about aggressive challenging behaviour?

2. As a PPIE member you have attended meetings, and commented on various topics – how have you found that? (prompt: what has been good about being part of the PETAL team?) Has it been what you expected? What could have been better?

3. Do you feel you have been encouraged and supported to give your views and experiences?

4. What do you think has been your most valuable contribution to the research so far? Can you give an example of how you have shared your experiences and how it has made a difference to the project?

5. Do you feel your contribution has been recognised and valued? Do you feel you have been treated as an equal partner? If so, in what way? (prompt: do you feel that your views are taken seriously and actioned?)

6. Have other members of the team made contributions that you think were also important? How was the relationship with other PPIE members, do you feel all members have had equal opportunities to contribute?

7. Do you think the discussions during PPIE meetings have informed decision making within the study?

8. Is there anything that you think we might not have considered when designing this study? Is there more research needed? What could have been better?

9. Was your role clear to you when you joined the study? How do you feel now about your role in the study?

10. In your opinion, what might be the reasons that researchers ask for your input?

11. Do you feel that the research team is approachable if you have any questions? Do they provide enough of their time to support you with your participation?

2B. Thank you for answering the questions on your involvement in the PETAL study, we are now going to move on to some questions about the meetings themselves.

12. Have you had any difficulties committing to the meetings due to time or other commitments? (prompts: have the meetings been arranged at an appropriate time and duration, with enough breaks etc?, what do you think about having meetings online?)

13. What do you think about the way the meetings are arranged and managed? (prompts: do you feel that you are provided with enough information, were your views heard and that you were able to express yourself during these meetings? What has made it easier for you to take part?)

14. Has the setting of the PPIE meetings taken your needs into consideration? (prompts: how do you feel about remote meetings and the forms of technology used?)

15. Do you feel that you have been reimbursed properly for your time and involvement? (prompts: Was this done promptly? Have you needed care costs covered? Have these been reimbursed?)

2C. Finally, I am going to ask you some questions about improvements we could make going forward.

16. Is there anything we can do to improve your involvement and others in the PETAL study? (prompt: what has not worked well?)

17. What would you like to see happen when the study finishes?

18. How do you think the PPIE group should contribute to sharing the findings? (dissemination- e.g. a PPIE journal paper, conference speakers etc)

19. Is there anything else you would like to add?

We have now come to the end of the interview questions for today. Thank you very much for your time.

# Appendix A: Topic guide for adults with a learning disability PPIE group

Hello! Thank you for your time today. We are going to ask you some questions about your experiences being involved as a member of the PETAL study Patient and Public Involvement and Engagement (PPIE) group. We would also like to understand if there is anything we can do to improve your involvement. All the information we collect will be kept confidential, and your answers will be anonymised. There is no pressure to answer every question. Please only answer questions you would like to answer.

We will have about 10 questions to ask. As everyone's left (researchers and facilitators), honestly feel free to answer as honestly as you like. It won't go back to them, and instead will just help to improve the study in the future. And like with everyone else that hopefully joins in the future, it will benefit them as well, as well as you, of course. Does anyone have any questions before we start?

1. Could you tell us about your experiences of helping the PETAL study
2. What do you think you have helped with the most so far?

1. How happy are you with your help towards this study?
2. Do you feel your views are heard and taken seriously by the researchers?
3. Do you feel that you are given enough help to be part of the study?
4. Do you hear enough from researchers?
5. What are some things that help you to take part in the meetings?
6. What are some things that make it harder for you to take part in meetings?
7. Do researchers need to do anything better?

1. Is there anything else you would like to add?

Does anyone have any questions they would like to ask?

We have now come to the end of the focus group questions for today. Thank you very much for your time.
